# Effect of the weight-loss program using daily self-weighing combined with personalized counseling led by village health volunteers in adults with obesity in a rural community, Thailand: a randomized controlled trial

**DOI:** 10.1186/s12875-023-02178-3

**Published:** 2023-10-28

**Authors:** Saharat Liampeng, Naphat Wongkliawrian, Surapas Junlawakkananon, Asaya Prapaso, Napatthawan Panichnantho, Saranphruk Kiengsiri, Maneepatsorn Sirisereewan, Onnalin Rungrotchanarak, Visavabhak Mahapol, Thanyaporn Boonsawat, Bhoom Tumrongteppitux, Pak Likitkulthanaporn, Sirakarn Tejavanija, Pongpisut Thakhampaeng, Mathirut Mungthin, Ram Rangsin, Boonsub Sakboonyarat

**Affiliations:** 1 Phramongkutklao College of Medicine, Bangkok, 10400 Thailand; 2 Department of Medicine, Phramongkutklao Hospital, Bangkok, 10400 Thailand; 3 Department of Military and Community Medicine, Phramongkutklao College of Medicine, Bangkok, 10400 Thailand; 4 Department of Parasitology, Phramongkutklao College of Medicine, Bangkok, 10400 Thailand

**Keywords:** Daily self-weighing, Body weight, Body mass index, Blood pressure, Rural community, Thailand

## Abstract

**Background:**

In a remote rural community in central Thailand, obesity prevalence among adults significantly rose from 33.9% in 2012 to 44.8% in 2018. Limited information on weight reduction studies in Thai rural communities was available. The present study aims to evaluate the effect of daily self-weighing combined with personalized counseling in order to reduce body weight (BW) and body mass index (BMI) as well as blood pressure (BP).

**Methods:**

A randomized controlled trial was carried out in a rural community in central Thailand.

One-hundred and seven adults were randomly allocated (1:2) to intervention and control groups. For 20 weeks, participants in the weight-loss program performed self-weighing twice daily and recorded their weight on the calendar. The program also offers weekly counseling visits by village health volunteers (VHV) who make home visits to participants. The primary outcomes were differences in mean change in BW at 20 weeks from baseline between the intervention and control groups.

**Results:**

A total of 107 participants were initially recruited. Of these, 36 participants were allocated to the intervention group and 57 participants to the control group. Significant differences in mean change in BW and BMI at the twelve-, sixteen-, and twenty-week follow-up from baseline between the two groups were observed. At twenty weeks, the mean change in BW was -1.2 kg (95% CI: -2.2, -0.3) and 0.3 kg (95% CI: -0.3, 0.8) in the intervention and control groups, respectively, with *p*-value = 0.007. Over 20 weeks of the study period, the estimated mean change in BW among the intervention group was 1.0 kg (95% CI -1.7, -0.2) lower than in the control group, with *p*-value = 0.015. Furthermore, changes in mean BMI and BP over the 20-week follow-up period in intervention participants were recognized.

**Conclusions:**

Our study demonstrates that daily self-weighing combined with personalized counseling led by VHV is feasible and can induce weight loss among adults with obesity in a rural community. In addition, the weight-loss program may be a promising additional tool for reducing BP.

**Trial registration:**

Trial identification number was TCTR20201020004; first submitted date: 20/10/2020.

**Supplementary Information:**

The online version contains supplementary material available at 10.1186/s12875-023-02178-3.

## Background

In 2016, the World Health Organization indicated that globally adults aged 18 years and older with body mass index (BMI) ≥ 25 kg/m^2^ totaled more than 1.9 billion; additionally, among them, over a million had BMI ≥ 30 kg/m^2^ [1]. In Thailand, recent studies emphasized that obesity prevalence was continuously rising in several populations. The National Health Examination Survey (NHES) reported that obesity prevalence among the Thai population aged ≥ 15 years increased from 37.5% in 2014 [2] to 42.2% in 2019 [3]. Similarly, the rising trends of obesity prevalence were noticed among military personnel [4, 5].

At present, epidemiologic transitions create several challenges to public health globally, including Thailand. For instance, the Thai population is considered one of the world’s promptly aged societies [6]. Furthermore, the magnitude of noncommunicable diseases (NCDs) in Thailand continuously increases and affects the health of people in urban and rural areas [2, 3, 7–9]. Obesity is an important risk factor causing NCDs encompassing type 2 diabetes (T2D), cancer, and cardiovascular diseases [10–13]. Therefore, reduction of weight and BMI is a crucial solution that is feasible and promises to alleviate the risk for NCDs [14–18].

Numerous randomized controlled trials showed that adults with obesity who participated in behavioral therapies focusing on self-monitoring for weighing [19, 20], dietary control [21], physical activity [22, 23], and other cognitive techniques, such as stimulus control or setting specific goals [24] significantly lost weight. However, a few studies indicated that the effect of self-monitoring intervention declined over time due to many difficulties in the adhesion of health promotion to self-control [25, 26]. Hopefully, community organizations play an essential role in improving health [27–29], such as encouraging weight reduction intervention in a rural setting [30].

Approximately 50% of Thai people reside in rural communities where the characteristics of healthcare resources and providers differ from those of urban areas [31]. In Thailand, since 1977, village health volunteers (VHV) have been the community health workers serving as the backbone of the healthcare delivery system and supporting the concept of community involvement and primary care unit (PCU) activities [32, 33]. Recently, Sakboonyarat et al. explored that obesity prevalence among adults in a remote rural community in central Thailand significantly climbed from 33.9% in 2012 to 44.8% in 2018 [34], which were compatible with the NHES reports [2, 3].

Regarding the existing evidence, the limitation of weight reduction study in Thai rural communities was available. Therefore, the weight-loss program using daily self-weighing combined with personalized counseling by VHV was established to support weight reduction. The present study desires to assess the effect of daily self-weighing combined with personalized counseling in order to reduce body weight (BW) and BMI as well as blood pressure (BP).

## Methods

### Study design and participants

A randomized controlled trial was implemented between November 2020 and March 2021 in the Na-Yao community, Tha Kradan Subdistrict, Sanam Chai Khet District, Chachoengsao Province (central Thailand, a remote rural area 180 km from Bangkok). Participants were recruited through an announcement at the Baan Na-Yao Health Promoting Hospital, a primary care unit located in the community. Adults between the ages of 18 and 60, a BMI of more than 27.5 kg/m^2^, a desire to participate in the trial, giving written informed consent, and a requirement to reduce BW were all required for eligibility. The exclusion criteria are described as follows:

- (i) being diagnosed with medical conditions that might affect weight,
- (ii) taking or expecting to receive medication that might affect weight,
- (iii) receiving diabetes treatment with either oral drugs or insulin injections,
- (iv) pregnant women,
- (v) having significant weight loss in the last six months,
- (vi) bedridden patients,
- (vii) history of substance use in the last six months,
- (viii) being diagnosed with coronary heart disease or cerebrovascular disease,
- (ix) history of antiobesity drug use,
- (x) participating in other clinical controlled trials.

A physician evaluated these criteria in baseline data collection or from the data in patients’ medical records. A total of 107 individuals were considered eligible. The information of the study was explained to the participants. All participants provided written informed consent to participate in the current study.

The estimated effect size of 1.4 kg, standard deviation (SD) of 2.0 kg, and the allocation ratio 1:2 was used to calculate the sample size with a power of 80% and a two-sided significance level of 95% [35]. The sample size resulted in 33 and 66 individuals in each group. In the present study, the participants would be randomly allocated (1:2) to intervention and control groups concerning the limitation of resources. Therefore, the sample size of 36 for the intervention group and 72 for the control group was finally estimated to mitigate the effect of possible losses during this trial.

### Randomization

**Fig. 1:**
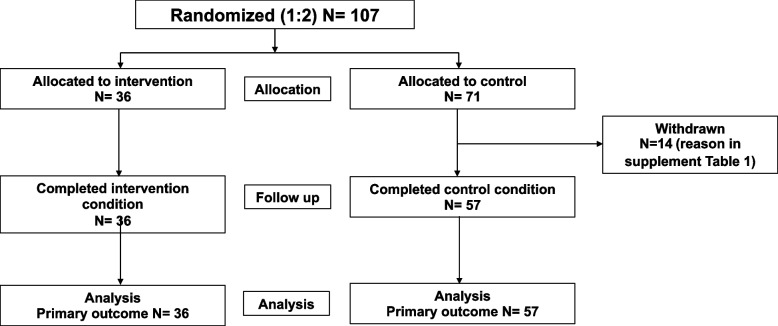
CONSORT participant flow diagram

The flow of study participants is presented in Fig. 1. One-hundred and seven adults were randomly allocated (1:2) to intervention and control groups through the use of simple randomization procedures involving computerized random numbers. Allocation data were generated by the staff of the data management unit, who had no contact with the investigators or participants, and were maintained at a secure central location until the completion of the baseline assessment. Treatment allocation was concealed until the time of randomization. All outcome data were kept until the final data entry for 20-week assessments was completed. Informed consent was withdrawn by 14 of 71 subjects from the control group. Therefore, the final distribution was 36 subjects (1 men and 35 women) in the intervention group and 57 subjects (2 men and 55 women) in the control group.

### Baseline assessment

The investigators who concealed the allocation conducted face-to-face interviews using standardized questionnaires conducted in October 2020 at Baan Na-Yao Health Promoting Hospital, Sanam Chai Khet District, Chachoengsao Province, to collect baseline information. The questionnaires contained demographic characteristics, such as age and sex, and comorbidities, like history of T2D, hypertension, and dyslipidemia. Height was measured through the use of a stadiometer (DETECTO, St. Webb City, MO, USA). BW was obtained from a body composition monitor (OMRON model HBF-212, Kyoto, Japan). Waist circumference (WC) was measured after full expiration using plastic tape at the umbilical level. BMI was calculated as weight in kilograms by height in meters squared. BP was measured by an operator trained in standardized technique following the 2019 Thai Treatment Guidelines of HT [36], through the use of an automatic BP monitor (OMRON model HEM-8712, Kyoto, Japan).

### Intervention

At baseline, the intervention group received health education about obesity and its complications. Moreover, knowledge of how to reduce their BW, including a hypocaloric diet and physical exercise, was also provided to the participants. Based on findings from the prior research of Willett et al., the following distribution of macronutrients was used to make dietary recommendations for the participants: A diet high in fiber and low in fat, as well as avoiding high-calorie drinks, was also advised. The recommended ratios for these factors were 40–45% carbohydrates, 30–35% fats, and 25–30% proteins [37]. Examples of hypocaloric food recipes, especially local food, also were given to participants. According to WHO guidelines on physical activity and sedentary behavior, physical exercise was recommended as moderate-intensity aerobic physical activity for 150–300 min per week or vigorous-intensity aerobic physical activity for 75–150 min per week [38]. The weight-loss program consisted of two main components: (1) daily self-weighing and (2) personalized counseling by trained VHV.

#### Daily self-weighing

For 20 weeks, participants in the intervention group weighed themselves twice daily (after waking up in the morning and before going to bed at night) using standardized digital scales. All participants were advised to wear nothing but undergarments to prevent the attachment of clothing weight to a BW measurement. In addition, a calendar for recording BW was given to illustrate the trend in the BW of individuals and raise awareness. After self-weighing, participants would record their BW on the calendar.

#### Personalized counseling

Well-trained VHV visited the participants at home once weekly for 20 weeks. Following the established procedure, the VHV engaged the participants during the home visit by greeting them and inquiring about their thoughts and any potential negative side effects of the weight-loss program. Additionally, the VHV also encouraged the participants to continue the hypocaloric diet following the example of a recipe, physical exercise, and daily self-weighing with a record. Furthermore, the VHV would notify the field research coordinators and registered nurses who worked at the Baan Na-Yao Health Promoting Hospital if adverse events were reported.

### Control

At baseline, the participants in the control group were engaged in the study to collect baseline measurements. Similarly, participants in the control group received health education about obesity and its complications and information about how to reduce their BW, entailing a hypocaloric diet and physical exercise, like the intervention group. Then, the outcomes in the control group were collected at four, eight, twelve, sixteen, and twenty weeks.

### Outcomes

BW is easy to measure and comprehend for evaluating the impact of the weight-loss program, particularly in rural settings. Therefore, the study’s primary outcomes were differences in mean change in BW at the twenty-week follow-up from baseline between the intervention and control groups. Secondary outcomes consisted of differences in mean changes in BMI, systolic blood pressure (SBP) and diastolic blood pressure (DBP) at the twenty-week follow-up from baseline between the two groups. In addition, a longitudinal study of the Thai NHES IV and V indicated that the Waist-to-Height ratio (WHtR) was a good predictor for all-cause and cardiovascular mortality [39]; hence, the differences in mean changes in WC and WHtR at twenty-week follow-up from baseline between the two groups would be evaluated.

### Ethics consideration

This study was reviewed and approved by the Royal Tai Army Medical Department Institutional Review Board. Written informed consent was obtained from all participants according to the WMA Declaration of Helsinki Ethics principles for medical research involving human subjects (approval number: R133h/63). Too, this study was registered in the Thai Clinical Trials Registry and obliged to disclose details of the 24 mandatory items of the WHO International Clinical Trials Registry Platform (Trial identification number was TCTR20201020004, first submitted date: 20/10/2020).

### Statistical analysis

Demographic data of participants were analyzed using descriptive statistics. The *chi*-square test was employed for categorical data. For continuous data with normality, the *t*-test was utilized; if there was nonnormality, Mann–Whitney U test would be exploited. The generalized estimating equations (GEE) method with vce(robust) option to get robust standard error was performed to investigate the outcome difference between the two groups over time. Regarding the primary outcome, the mean differences in BW at the twenty-week follow-up and at baseline, between intervention and control groups were compared. In addition, the GEE method was used to estimate the difference in the evolution of BW with respect to baseline over a 20-week follow-up between two groups. The secondary outcomes, including BMI, WC, WHtR, SBP, and DBP, were also analyzed as the BW.

Furthermore, the GEE method also was applied to compare the outcome measurements, enclosing BW, BMI, WC, WHtR, SBP, and DBP, within groups at baseline and follow-up periods, and calculate the *p*-value for the trend of outcomes in intervention and control groups. All statistical significance was considered for a two-sided *p*-value less than 0.05. Statistical analyses were performed using StataCorp, 2021, *Stata Statistical Software: Release 17,* College Station, TX, USA: StataCorp LLC.

In addition, we performed a sensitivity analysis using the GEE method to adjust the age and sex of study participants to estimate the difference in the evolution of BW with respect to baseline over a 20-week follow-up.

## Results

### Baseline characteristics

A total of 107 participants were randomized. There were 36 intervention and 71 control participants (CONSORT: Fig. 1). Of these, 14 control participants were withdrawn for reasons shown in Supplementary Table 1. Of the total 93 participants in the study (excluding 14 withdrawals), 36 intervention participants and 57 control participants completed the study. Table 1 presents the baseline characteristics of participants. The mean age of intervention participants was 43.1 ± 9.9 years (range: 18–60 years), while the mean age of control participants was 44.8 ± 10.6 years (range: 18–59 years). In terms of BW, the mean in the intervention group was 79.7 ± 11.2 kg (range: 65.3–110.0 kg), like the mean in the control group was 79.4 ± 11.0 kg (range: 56.2–119.6 kg). At baseline, the mean BMI of intervention participants was 32.4 ± 3.5 kg/m^2^ (range: 28.2–42.8 kg/m^2^), like the mean BMI of control participants which was 32.5 ± 3.3 kg/m^2^ (range: 27.2–44.5 kg/m^2^). Regarding WC, the mean in the intervention group was 100.1 ± 9.3 cm., and the mean in the control group was 101.2 ± 10.1 cm. For WHtR, the mean in the intervention group was 0.64 ± 0.06, like the mean in the control group was 0.65 ± 0.06.

**Table 1:** Baseline characteristics of study participants

Baseline characteristics*	Intervention	Control	Withdraw (from control)
	***n* = 36**	***n* = 57**	***n* = 14**
**Sex**			
Male	1 (2.8)	2 (3.5)	5 (35.7)
Female	35 (97.2)	55 (96.5)	9 (64.3)
**Age (years)**			
Mean ± SD	43.1 ± 9.9	44.8 ± 10.6	45.5 ± 9.5
Median (min–max)	44.0 (18.0–60.0)	46.0 (18.0–59.0)	47.0 (30.0–59.0)
**Diabetes mellitus**			
No	35 (97.2)	53 (93.0)	12 (85.7)
Yes	1 (2.8)	4 (7.0)	2 (14.3)
**Hypertension**			
No	22 (61.1)	42 (73.7)	10 (71.4)
Yes	14 (38.9)	15 (26.3)	4 (28.6)
**Dyslipidemia**			
No	32 (88.9)	50 (87.7)	12 (85.7)
Yes	4 (11.1)	7 (12.3)	2 (14.3)
**Body weight (kg)**			
Mean ± SD	79.7 ± 11.2	79.4 ± 11.0	77.7 ± 10.4
Median (min–max)	78.0 (65.3–110.0)	78.6 (56.2–119.6)	80.0 (59.0–94.0)
**Body mass index (kg/m**^**2**^**)**			
Mean ± SD	32.4 ± 3.5	32.5 ± 3.3	31.4 ± 2.0
Median (min–max)	31.4 (28.2–42.8)	32.0 (27.2–44.5)	31.2 (27.5–35.4)
**Waist circumference (cm)**			
Mean ± SD	100.1 ± 9.3	101.2 ± 10.1	92.7 ± 7.8
Median (min–max)	99.5 (76.0–121.0)	102.0 (76.0–135.0)	95.0 (81.0–105.0)
**Waist to height ratio**			
Mean ± SD	0.64 ± 0.06	0.65 ± 0.06	0.59 ± 0.06
Median (min–max)	0.64 (0.51–0.76)	0.65 (0.48–0.82)	0.60 (0.52–0.68)
**Systolic blood pressure (mmHg)**			
Mean ± SD	131.6 ± 19.3	127.6 ± 15.8	124.7 ± 18.7
Median (min–max)	128.8 (97.0–176.5)	125.5 (93.5–168.0)	116.0 (106.0–164.0)
**Diastolic blood pressure (mmHg)**			
Mean ± SD	84.5 ± 13.5	79.9 ± 10.6	81.3 ± 9.2
Median (min–max)	83.8 (55.0–124.0)	80.0 (53.5–105.5)	82.0 (64.5–95.5)

*SD* Standard deviation

^*^No statistically significant differences were observed between the intervention and control groups regarding all baseline characteristics (*p*-value > 0.05)

### Effect of the weight-loss program

Table 2 illustrates the outcomes within groups at baseline and follow-up periods. Among intervention participants, significant mean BW decreased from 79.7 ± 11.2 kg at baseline to 78.4 ± 12.0 kg at the twenty-week follow-up (*p*-value < 0.05). Similarly, significant mean BMI dropped from 32.4 ± 3.5 kg/m^2^ at baseline to 31.9 ± 3.8 kg/m^2^ at twenty weeks (*p*-value < 0.05). Over the twenty-week follow-up period, significant changes in mean BW and BMI were observed (*p* for trend = 0.011 and 0.009, respectively). In contrast, a significant change in mean WC and mean WHtR was not found. The mean SBP at baseline was 131.6 ± 19.3 mmHg and diminished to 126.8 ± 16.9 mmHg at the 20-week follow-up (*p* for trend = 0.077). The mean DBP at baseline was 84.5 ± 13.5 and declined to 81.2 ± 11.2 mmHg at the 20-week follow-up (*p* for trend = 0.191).

**Table 2:** Comparing outcome measurements between the intervention group and control group at follow-up periods

**Outcomes**	**Baseline**	**4-week**	**8-week**	**12-week**	**16-week**	**20-week**	***p* for trend**^b^
	**mean ± SD**	**mean ± SD**	**mean ± SD**	**mean ± SD**	**mean ± SD**	**mean ± SD**	
**Body weight (kg)**							
Intervention	79.7 ± 11.2	79.0 ± 11.5*	78.5 ± 11.7*	78.6 ± 11.7*	78.7 ± 12.0*	78.4 ± 12.0*	0.011
Control	79.4 ± 11.0	79.0 ± 10.9	78.9 ± 10.6*	79.6 ± 11.2	79.7 ± 11.2	79.6 ± 11.3	0.347
*p*-value^a^	0.904	0.991	0.866	0.677	0.692	0.630	
**Body mass index (kg/m**^**2**^**)**							
Intervention	32.4 ± 3.5	32.2 ± 3.5*	32.0 ± 3.6*	32.0 ± 3.6*	32.0 ± 3.8*	31.9 ± 3.8*	0.009
Control	32.5 ± 3.3	32.4 ± 3.3	32.3 ± 3.3*	32.6 ± 3.5	32.6 ± 3.4	32.6 ± 3.5	0.354
*p*-value^a^	0.917	0.792	0.616	0.415	0.436	0.381	
**Waist circumference (cm)**							
Intervention	100.1 ± 9.3	99.1 ± 10.0	99.1 ± 8.9	98.8 ± 8.3	100.6 ± 10.8	99.7 ± 11.5	0.802
Control	101.2 ± 10.1	101.5 ± 8.3	99.4 ± 8.4*	101.1 ± 9.6	100.5 ± 9.2	100.6 ± 9.3	0.474
*p*-value^a^	0.598	0.234	0.843	0.203	0.976	0.700	
**Waist to height ratio**							
Intervention	0.64 ± 0.06	0.63 ± 0.06	0.63 ± 0.06	0.63 ± 0.05	0.64 ± 0.06	0.64 ± 0.07	0.781
Control	0.65 ± 0.06	0.65 ± 0.05	0.64 ± 0.05*	0.65 ± 0.06	0.64 ± 0.06	0.64 ± 0.06	0.470
*p*-value^a^	0.472	0.172	0.736	0.140	0.888	0.566	
**Systolic blood pressure (mmHg)**							
Intervention	131.6 ± 19.3	121.9 ± 16.0*	126.6 ± 19.5*	123.9 ± 17.9*	127.4 ± 20.3	126.8 ± 16.9	0.077
Control	127.6 ± 15.8	123.5 ± 13.2*	126.4 ± 14.4	125.8 ± 13.0	125.0 ± 17.1	125.2 ± 15.6	0.291
*p*-value^a^	0.292	0.618	0.958	0.587	0.541	0.644	
**Diastolic blood pressure (mmHg)**							
Intervention	84.5 ± 13.5	77.6 ± 12.8*	82.2 ± 15.9	80.6 ± 12.5*	84.3 ± 15.4	81.2 ± 11.2	0.191
Control	79.9 ± 10.6	77.6 ± 8.1	79.4 ± 8.8	80.7 ± 8.5	80.1 ± 8.5	78.0 ± 10.1	0.423
*p*-value^a^	0.082	0.998	0.326	0.964	0.137	0.171	

*SD* Standard deviation

^*^p-value < 0.05 when comparing with baseline

^a^Comparison between intervention group and control group

^b^The generalized estimating equations (GEE) method with robust standard error

In the control group, the mean BW was 79.4 ± 11.0 kg and 79.6 ± 11.3 kg at baseline and twenty weeks, respectively. The mean BMI was 32.5 ± 3.3 kg/m^2^ at baseline and 32.6 ± 3.5 kg/m^2^ at twenty weeks. In terms of WC, WHtR, SBP, and DBP, no differences were discovered from the baseline to the 20-week follow-up.

The mean BMI, BW, WC, and WHtR of participants in intervention and control groups did not vary at the follow-up of four, eight, twelve, sixteen, and twenty weeks. In addition, no difference was remarked in mean SBP and DBP among participants during the same periods of follow-up.

### Primary outcomes

**Fig. 2:**
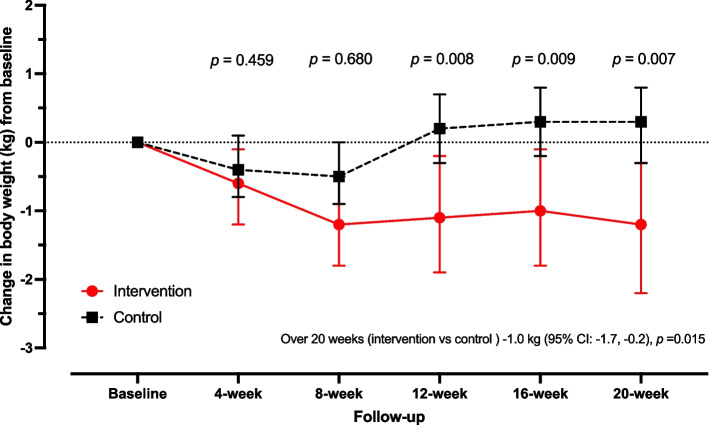
Comparison of mean change of body weight (kg) at follow-up periods from baseline between the intervention group and control group

Table 3 compares the differences in mean change in outcomes at follow-up periods from baseline between the intervention group and the control group. Significant differences in mean change in BW and BMI at the twelve-, sixteen-, and twenty-week follow-up from baseline between the two groups were recognized. At twenty weeks, the mean change in BW was -1.2 kg (95% CI: -2.2, -0.3) and 0.3 kg (95% CI: -0.3, 0.8) in the intervention and control groups, respectively, with *p*-value = 0.007 (Fig. 2). Over 20 weeks of the study period, the estimated mean change in BW among the intervention group was 1.0 kg (95% CI -1.7, -0.2) lower than in the control group, with *p*-value = 0.015 (Table 3).

**Table 3:** Comparing differences of mean change in outcomes at follow-up periods from baseline between the intervention group and control group

**Outcomes**	**4-week**	**8-week**	**12-week**	**16-week**	**20-week**	**Over 20 weeks**^b^
	**mean change (95% CI)**	**mean change (95% CI)**	**mean change (95% CI)**	**mean change (95% CI)**	**mean change (95% CI)**	**Difference mean change (95%CI)**
**Primary outcome**						
**Body weight (kg)**						
Intervention	-0.6 (-1.2, -0.1)	-1.2 (-1.8, -0.5)	-1.1 (-1.9, -0.2)	-1.0 (-1.8, -0.1)	**-1.2 (-2.2, -0.3)**	-1.0 (-1.7, -0.2)
Control	-0.4 (-0.8, 0.1)	-0.5 (-0.9, 0.0)	0.2 (-0.3, 0.7)	0.3 (-0.2, 0.8)	**0.3 (-0.3, 0.8)**	Ref
*p*-value^a^	0.459	0.680	0.008	0.009	**0.007**	0.015
**Secondary outcomes**						
**Body mass index (kg/m**^**2**^**)**						
Intervention	-0.3 (-0.5, 0.0)	-0.5 (-0.7, -0.2)	-0.5 (-0.8, -0.1)	-0.4 (-0.8, -0.1)	-0.5 (-0.9, -0.1)	-0.4 (-0.7, -0.1)
Control	-0.3 (-0.5, 0.0)	-0.2 (-0.4, 0.0)	0.1 (-0.1, 0.3)	0.1 (-0.1, 0.3)	0.1 (-0.1, 0.3)	Ref
*p*-value^a^	0.401	0.052	0.007	0.007	0.006	0.012
**Waist circumference (cm)**						
Intervention	-1.0 (-3.3, 1.4)	-1.1 (-3.4, 1.2)	-1.4 (-3.4, 0.6)	0.4 (-1.9, 2.7)	-0.4 (-3.0, 2.1)	-0.3 (-2.5, 1.9)
Control	0.3 (-1.5, 2.1)	-1.8 (-3.4, -0.1)	-0.1 (-2.0, 1.8)	-0.7 (-2.5, 1.1)	-0.6 (-2.4, 1.2)	Ref
*p*-value^a^	0.374	0.614	0.335	0.434	0.900	0.805
**Waist to height ratio**						
Intervention	-0.01 (-0.02, 0.01)	-0.01 (-0.02, 0.01)	-0.01 (-0.02, 0.00)	0.00 (-0.01, 0.02)	0.00 (-0.02, 0.01)	0.00 (-0.02, 0.01)
Control	0.00 (-0.01, 0.01)	-0.01 (-0.02, 0.00)	0.00 (-0.01, 0.01)	0.00 (-0.02, 0.01)	0.00 (-0.02, 0.01)	Ref
*p*-value^a^	0.374	0.578	0.346	0.445	0.920	0.807
**Systolic blood pressure (mmHg)**						
Intervention	-9.7 (-13.8, -5.5)	-5.0 (-8.9, -1.1)	-7.7 (-11.5, -3.8)	-4.1 (-8.7, 0.4)	-4.8 (-9.9, 0.4)	-3.9 (-8.6, 0.8)
Control	-4.1 (-7.9, -0.2)	-1.2 (-5.2, 2.8)	-1.8 (-6.0, 2.4)	-2.6 (-7.1, 1.9)	-2.3 (-6.2, 1.5)	Ref
*p*-value^a^	0.046	0.169	0.036	0.626	0.446	0.103
**Diastolic blood pressure (mmHg)**						
Intervention	-7 (-10.3, -3.6)	-2.3 (-6.2, 1.7)	-3.9 (-7.2, -0.7)	-0.2 (-3.8, 3.3)	-3.3 (-7.5, 0.8)	-2.7 (-6.4, 0.9)
Control	-2.4 (-5.1, 0.3)	-0.5 (-3.7, 2.6)	0.7 (-2.6, 4.1)	0.2 (-2.5, 2.9)	-1.9 (-4.8, 1.1)	Ref
*p*-value^a^	0.030	0.482	0.041	0.844	0.560	0.145

*CI* Confidence interval

^a^Comparison between intervention group and control group

^b^The generalized estimating equations (GEE) method with robust standard error

### Secondary outcomes

**Fig. 3:**
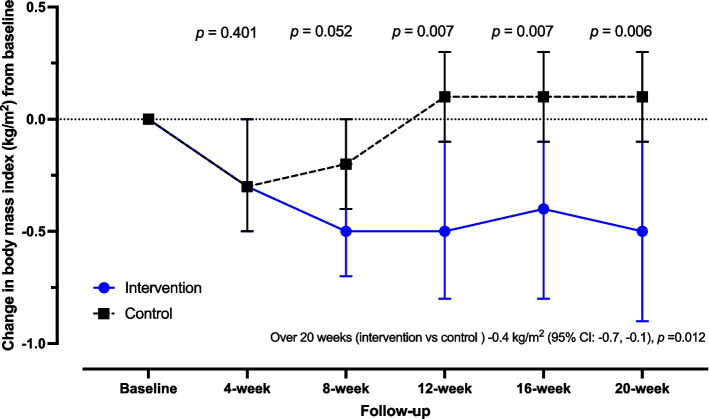
Comparison of mean change of body mass index (kg/m^2^) at follow-up periods from baseline between the intervention group and control group

At twenty weeks, the mean change in BMI was -0.5 kg/m^2^ (95% CI: -0.9, -0.1) in the intervention group and 0.1 kg/m^2^ (95% CI: -0.1, 0.3) in the control group, with *p*-value = 0.006 (Fig. 3). For both WC and WHrt, there was no difference in mean change at a twenty-week follow-up from baseline between intervention and control groups (Table 3). In terms of BP, there was no significant difference in mean change in BP at a twenty-week follow-up from baseline between intervention and control groups (Table 3). At twenty weeks, the mean change in SBP was -4.8 mmHg (95% CI: -9.9, 0.4) and -2.3 mmHg (95% CI: -6.2, 1.5) in the intervention and control groups, respectively, with *p*-value = 0.446. The mean change in DBP was -3.3 mmHg (95% CI: -7.5, 0.8) in the intervention group and -1.9 mmHg (95% CI: -4.8, 1.1) in the control group, with *p*-value = 0.560. Adverse events, such as palpitation, fainting, dizziness, trembling, and being weak with hunger, were not reported among participants over the twenty-week follow-up.

Regarding the sensitivity analysis, the GEE method accounting for age and sex revealed that the estimated mean change in BW among the intervention group was -0.9 kg (95% CI -1.7, -0.2) lower than in the control group, with *p*-value = 0.012, over twenty weeks of the study period (Supplementary Table 2) relatively followed the same pattern as the primary analysis.

## Discussion

To our knowledge, this constitutes the first study, a randomized controlled trial, that assesses the effect of daily self-weighing combined with personalized counseling for weight reduction in a rural community in Thailand. We found that, after twenty weeks of follow-up, there was a statistically significant effect on BW and BMI reduction among participants who received a weight-loss program. Furthermore, the outcome suggested that, at 20 weeks of follow-up, the weight-loss program facilitated a greater reduction in BW and BMI in the intervention group in comparison with the control group. In contrast, the weight-loss program had no effect on the secondary outcome of BP reduction.

For 20 weeks, participants in the weight-loss program performed self-weighing twice daily and recorded their weight on the calendar. The program also provides VHV visiting the participants at home once weekly. Therefore, intervention participants could see the trends in their BW over follow-up time which were recorded on the calendar. Furthermore, the VHV home visit may facilitate the maintenance of a hypocaloric diet and daily self-weighing with participants’ records for 20 weeks.

Robust evidence confirmed that regular self-weighing was associated with more weight loss [40–42]. Moreover, the related study also indicated that individuals lost more BW with daily and weekly weighing than with monthly weighing [43]. In the present study, the BW records twice daily on the calendar may be the additional tool that illustrates the trends in BW to individuals. Participants can compare their BW between today and yesterday as well as other days. Regarding the Health Action Process Approach [44] and Carver and Scheier’s Control Theory [45], this tool may enhance the reflective process contributing to health behavior maintenance, such as a hypocaloric diet which is one of the components of the weight-loss program [46].

Nevertheless, some related studies revealed that the effect of self-monitoring might decline over time [26]. The present study detected a greater decrease in BW and BMI in intervention participants at 12-, 16-, and 20-week follow-ups. The well-trained VHV of the weight-loss program visiting the intervention participants at home weekly may simplify the maintenance of a hypocaloric diet and self-weighing with records. The related studies in Thailand and the UK may support this finding that well-trained providers act as social supporters to improve health outcomes [27, 47]. The VHV in the weight-loss program in this study stand for the community health workers who support healthcare providers at the local primary care units in rural communities. However, given the burden of providing care for so many different people in the community, there are currently insufficient human resources. Thus, in the future, an innovative network of homecare providers (WinCare), novel human resources in the community who were not VHV on duty formerly [27], may perform this function rather than VHV.

Our results reported that there was no significant difference in mean change in BP at a twenty-week follow-up from baseline between intervention and control groups. However, a decrease in SBP and DBP over a 20-week follow-up among intervention participants was pinpointed, which is compatible with the related study in Thai patients with NCDs [27]. In the present study, the VHV also explored the adverse psychological and physical effects related to the weight-loss program. No adverse events were reported among participants in this study that corresponded to the related reports [40–42]. Thus, the weight-loss program, like daily self-weighing combined with personalized counseling, can be an effective and safe strategy that should be implemented in weight control programs among adults with high BMI residing in rural areas and may also be a promising additional tool for reducing BP.

Being a community-based randomized control trial was one of this study’s advantages. In addition, the participants were adults residing in a rural community. Hence, the results are robust for the adults with obesity in the rural areas, which is the residential area of half of the Thai population.

Regarding the study’s drawbacks, the first was its small sample size and uneven randomized allocation (2:1), which might have an impact on the power of study. Second, most study participants were females; thus, the results of our study could not be generalized to males. Third, 14 control participants withdrew from the study, which may affect the balance of baseline characteristics after allocation. However, no statistically significant differences were illustrated between the intervention and control groups regarding all baseline characteristics. Further, we also performed a sensitivity analysis to adjust sex and age in the GEE method; the results were relatively the same as the primary analysis. Fourth, due to a lack of resources, we were unable to examine the blood and urine samples for lipid profiles and plasma glucose as the secondary outcomes; furthermore, urine sodium for assessing sodium consumption which may affect BW and BP also was not evaluated. Fifth, in the present study, the total energy intake and physical activities were not assessed during the study period, which may influence the study outcomes. Further research needs to evaluate whether dietary intake and physical activities improve the actual evaluation of the effect of intervention. Finally, the present study was conducted between November 2020 and March 2021, covering the new year festival, which may affect eating behavior and study outcomes among study participants. Also, the study period coincided with the COVID-19 pandemic which only had an impact on Bangkok and Samut Sakhorn province in Thailand. On the other hand, the rural town of Nayao in Chacheongsao province was unaffected. However, the COVID-19 pandemic may have some impacts on the daily life activities of people. In the future, a more extensive community-based randomized control trial should be investigated to examine the effects of an intervention on the other body compositions, such as muscle mass, visceral fat, and body fat, as well as laboratory results, like lipid profile and plasma glucose.

## Conclusion

The present study emphasized that daily self-weighing combined with personalized counseling was feasible and could induce significant weight loss among adults with high BMI in a rural community. The weight-loss program may also be a promising additional tool for reducing BP. Further, the effect of intervention on other body compositions and metabolic parameters should be investigated.

### Supplementary Information

**Additional file 1: Supplement Table 1**. Reasons for participant withdrawal from study. **Supplementary Table 2.** Comparing differences of mean change in outcomes at follow-up periods from baseline between the intervention group and control group.

## Data Availability

The datasets generated or analyzed during the current study are not publicly available because the data sets contain confidential information. Thus, due to ethics restrictions concerning the data sets, they are available from the corresponding author upon reasonable request.

## Abbreviations

NCD: Noncommunicable diseases
HT: Hypertension
T2D: Type 2 diabetes
BW: Body weight
BMI: Body mass index
WC: Waist circumference
WHtR: Waist to height ratio
BP: Blood pressure
SBP: Systolic blood pressure
DBP: Diastolic blood pressure
VHV: Village health volunteers
