# Supplementary material for: Effect of the weight-loss program using daily self-weighing combined with personalized counseling led by village health volunteers in adults with obesity in a rural community, Thailand: a randomized controlled trial
Source: BMC Prim Care. 2023 Oct 28;24:226. doi: 10.1186/s12875-023-02178-3 (PMC10612287; doi:10.1186/s12875-023-02178-3)
Supplement: Supplementary file 1 — Additional file 1: Supplement Table 1. Reasons for participant withdrawal from study. Supplementary Table 2. Comparing differences of mean change in outcomes at follow-up periods from baseline between the intervention group and control group. [file 12875_2023_2178_MOESM1_ESM.docx]

**Additional file**

**Effect of the Weight-Loss Program Using Daily Self-Weighing Combined with Personalized Counseling Led by Village Health Volunteers in Adults with Obesity in a Rural Community, Thailand: A Randomized Controlled Trial**

Saharat Liampeng^[1](https://orcid.org/0000-0001-7866-9650)^, Naphat Wongkliawrian^1^, Surapas Junlawakkananon^1^, Asaya Prapaso^1^, Napatthawan Panichnantho^1^, Saranphruk Kiengsiri^1^, Maneepatsorn Sirisereewan^1^, Onnalin Rungrotchanarak^1^, Visavabhak Mahapol^1^, Thanyaporn Boonsawat^1^, Bhoom Tumrongteppitux^1^, Pak Likitkulthanaporn^1^, Sirakarn Tejavanija^2^, Pongpisut Thakhampaeng^3^, , Mathirut Mungthin^4^, *Ram Rangsin^3^, *Boonsub Sakboonyarat^3^

^1^Phramongkutklao College of Medicine, Bangkok 10400, Thailand

^2^Department of Medicine, Phramongkutklao Hospital, Bangkok 10400, Thailand

^3^Department of Military and Community Medicine, Phramongkutklao College of Medicine, Bangkok 10400, Thailand

^4^Department of Parasitology, Phramongkutklao College of Medicine, Bangkok 10400, Thailand

^*^**Corresponding author**:

**Ram Rangsin, M.D., M.P.H., Dr.P.H.**

Department of Military and Community Medicine, Phramongkutklao College of Medicine, Bangkok 10400, Thailand

Email: r_rangsin@yahoo.com

**Boonsub Sakboonyarat, M.D., M.P.H.**

Department of Military and Community Medicine, Phramongkutklao College of Medicine, Bangkok 10400, Thailand

Email: boonsub1991@pcm.ac.th

**Supplement Table 1**. Reasons for participant withdrawal from study

| **Reason for withdrawal** | **N** | **Withdrawn by** |
| --- | --- | --- |
| Felt uncomfortable participating every four weeks for a 20-week follow-up; Withdrew after randomization but before starting the study. | 13 | Participants |
| Moved away from the community; Withdrew after randomization but before 4 week follow-up | 1 | Investigator |

**Supplementary Table 2.** Comparing differences of mean change in outcomes at follow-up periods from baseline between the intervention group and control group.

| **Outcomes** | **Over 20 weeks**^§^ |
| --- | --- |
|  | **Difference mean change (95%CI)** |
| **Primary outcome** |  |
| **Body weight (kg)** |  |
| Intervention | -0.9 (-1.7, -0.2) |
| Control | Ref. |
| *p*-value^†^ | 0.012 |
| **Secondary outcomes** |  |
| **Body mass index (kg/m^2^)** |  |
| Intervention | -0.4 (-0.7, -0.1) |
| Control | Ref. |
| *p*-value^†^ | 0.009 |
| **Waist circumference (cm)** |  |
| Intervention | -0.4 (-2.6, 1.8) |
| Control | Ref. |
| *p*-value^†^ | 0.691 |
| **Waist to height ratio** |  |
| Intervention | 0.00 (-0.02, 0.01) |
| Control | Ref. |
| *p*-value^†^ | 0.691 |
| **Systolic blood pressure (mmHg)** |  |
| Intervention | -4.3 (-8.8, 0.3) |
| Control | Ref. |
| *p*-value^†^ | 0.064 |
| **Diastolic blood pressure (mmHg)** |  |
| Intervention | -2.9 (-6.5, 0.8) |
| Control | Ref. |
| *p*-value^†^ | 0.124 |

CI: confidence interval, Ref: reference

^§^The general estimating equations (GEE) method with robust standard error (adjusting for age and sex)
